# Phosphorus Dendrimers as Nanotools against Cancers†

**DOI:** 10.3390/molecules25153333

**Published:** 2020-07-22

**Authors:** Anne-Marie Caminade

**Affiliations:** 1 Laboratoire de Chimie de Coordination du CNRS, UPR 8241, 205 Route de Narbonne, BP 44099, 31077 Toulouse CEDEX 4, France; anne-marie.caminade@lcc-toulouse.fr; 2 LCC-CNRS, Université de Toulouse, CNRS, Toulouse, France

**Keywords:** dendrimers, phosphorhydrazone, anticancer drugs, transfection, metal complexes, oligonucleotides

## Abstract

This review concerns the use of dendrimers, especially of phosphorhydrazone dendrimers, against cancers. After the introduction, the review is organized in three main topics, depending on the role played by the phosphorus dendrimers against cancers: (i) as drugs by themselves; (ii) as carriers of drugs; and (iii) as indirect inducer of cancerous cell death. In the first part, two main types of phosphorus dendrimers are considered: those functionalized on the surface by diverse organic derivatives, including known drugs, and those functionalized by diverse metal complexes. The second part will display the role of dendrimers as carriers of anticancer “drugs”, which can be either small molecules or anticancer siRNAs, or the combination of both. In the third part are gathered a few examples of phosphorhydrazone dendrimers that are not cytotoxic by themselves, but which under certain circumstances induce a cytotoxic effect on cancerous cells. These examples include a positive influence on the human immune system and the combination of bioimaging with photodynamic therapy properties.

## 1. Introduction

**Figure 1:**
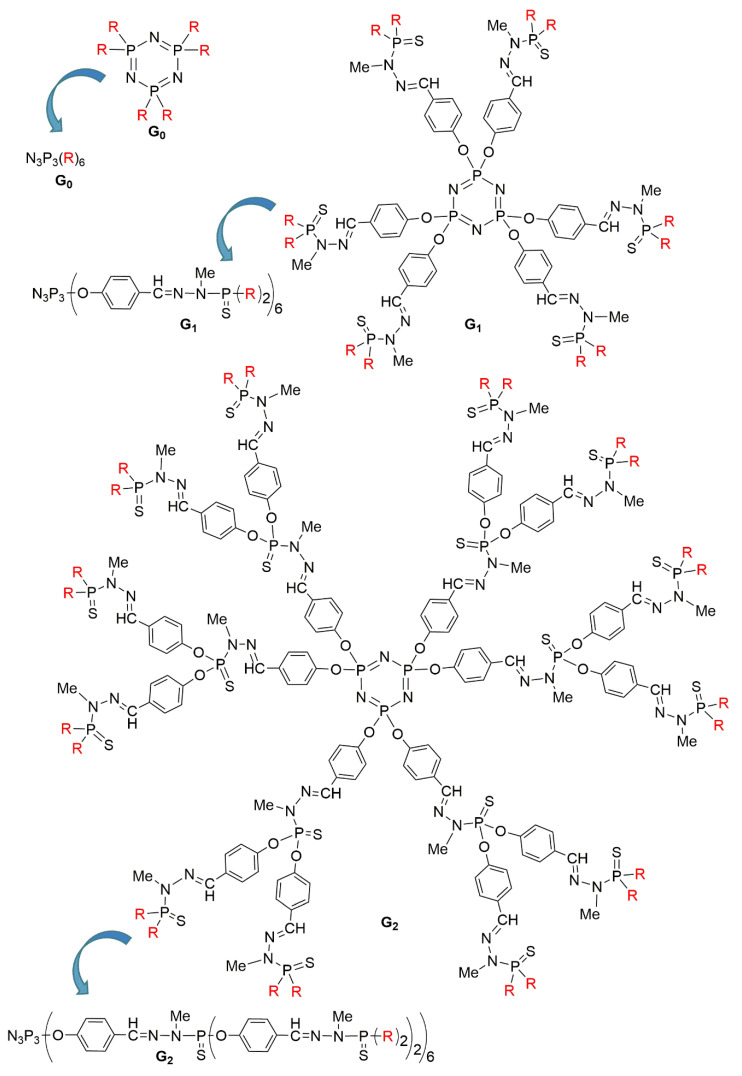
Full chemical structure of phosphorhydrazone dendrimers of generations zero (**G_0_**), one (**G_1_**), and two (**G_2_**). For each dendrimer, the same structure is shown in a linear form with parentheses at each level of branching. The linear structure will be used in all the following Figures.

The concept of “branching” at the macromolecular level dates back to the 1940s, with theory and experiments regarding hyperbranched polymers [1,2]. Dendrimers are macromolecules of a few nanometers size, constituted of branched identical units arranged around a central core. They are also called molecular trees, to describe their structure, and contrarily to hyperbranched polymers, dendrimers are not synthesized by polymerization reactions, but step-by-step. Each level of branching layer is called a generation. The higher the generation number is, the larger the dendrimer is. Since the pioneering works in the late 1970s [3] and the early 1980s [4], the area of dendrimers has been blossoming, and a lot of astonishing properties have been reported in diverse fields [5]. Most dendrimers are based on organic branches, joined by nitrogen atoms at the branching points. The well-known PAMAM (polyamidoamine) [6] dendrimers pertain to this category, as well as the PPI (polypropyleneimine) [7] dendrimers. However, other types of dendrimers having “inorganic” elements as branching points [8], such as silicon [9] or phosphorus [10] have been synthesized also very early [11]. Among these inorganic dendrimers, two families have emerged: carbosilane dendrimers [12], and phosphorhydrazone dendrimers [11]. The latter were synthesized first up to generation 4 [13], then up to generation 7 [14], then generation 10 [15], and finally generation 12 [16], which has been the highest generation for over 20 years among all types of dendrimers. These first experiments were carried with a trifunctional core based on P(S)Cl_3_, but later on, most of the experiments were carried out with the hexafunctional core based on the cyclotriphosphazene N_3_P_3_Cl_6_ [17], as illustrated in Figure 1.

These dendrimers have generated hundreds of publications, first concerning the synthesis of very original dendritic structures [18], then for their use as catalysts [19], for elaborating nanomaterials [20], and in biology or nanomedicine [21,22]. Among the numerous properties of phosphorus dendrimers in the latter fields, one can cite their use against prion diseases [23]; against Alzheimer disease [24]; against HIV [25,26], against rheumatoid arthritis [27], uveitis [28], lung inflammation [29]; and against various types of cancers, which will be the main topic of this review.

This review will be organized depending on the role played by the phosphorus dendrimers against cancers, either as drugs by themselves, as carriers of drugs, or as indirect inducers of cancerous cell death. 

## 2. Phosphorus Dendrimers as Anticancer Drugs by Themselves

In this section we will consider two main types of phosphorus dendrimers: those functionalized on the surface by diverse organic derivatives, including known drugs, and those functionalized by metal complexes. 

### 2.1. Phosphorhydrazone Dendrimers Functionalized by Organic Derivatives 

**Figure 2:**
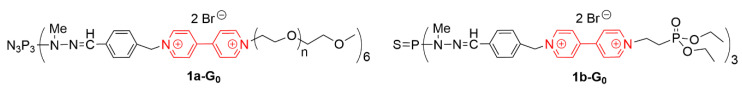
Small viologen dendrimers having anticancer properties.

A series of small viologen phosphorus dendrimers was synthesized [30], and was found to have a relatively low toxicity [31]. Amongst these compounds, two of them displayed interesting anticancer properties in vitro (Figure 2) [32]. These compounds do not bear known anticancer drugs on their surface or structure. Compound **1a-G_0_** has practically no deleterious activity against red blood cells (about 3% hemolysis after 24 h at 20 µMol concentration), it is not harmful for B14 Chinese hamster peritoneal fibroblasts (non-cancerous cells) and has practically no effect against diverse types of micro-organisms, but **1a-G_0_** is able to decrease the viability of N2a cells (fast growing mouse neuroblastoma cell line) to less than 50% at 20 µMol. The small viologen dendrimer **1b-G_0_** is built from the trifunctional P(S)Cl_3_ core instead of the hexafunctional cyclotriphosphazene core, used for the synthesis of **1a-G_0_**, and it bears three phosphonate terminal functions. This compound **1b-G_0_** is relatively toxic against red blood cells (about 10% hemolysis after 24 h at 20 µMol concentration); it is not harmful toward B14 fibroblasts, but has a certain activity against *S. aureus* and *E. Coli*, while being toxic to N2a cells (viability is about 30% at 20 µMol).

**Figure 3:**
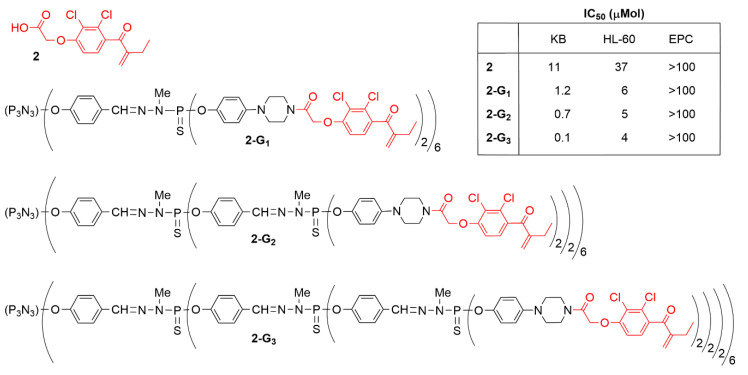
Ethacrynic acid (**2**), and generations 1 to 3 of phosphorhydrazone dendrimers functionalized with ethacrynic acid. IC_50_ values (in micromols) against two cancerous cell lines (KB and HL-60), and one non-cancerous (EPC).

Ethacrynic acid (compound **2** in Figure 3) is a diuretic used in the treatment of high blood pressure and swelling [33]. It has also been shown to inhibit cell growth and induce apoptosis in several cancer cell lines at high concentrations (30–50 μM) [34]. It is an inhibitor of glutathione transferase [35] and has been tested in particular against multiple myeloma, including as adjuvant in clinical trials [36]. A series of modifications of ethacrynic acid have been carried out to determine which part of the molecule, acid or alkene could be modified for enabling the grafting to dendrimers, without decreasing the anti-cancer efficiency. More than 25 derivatives of ethacrynic acid have been synthesized in this study [37]. Two key points have been demonstrated: (i) when the alkene bond of ethacrynic acid is modified, the anti-proliferative activity is totally lost; (ii) addition of a lateral chain through the carboxylic acid moiety results in a drastic increase of the anti-proliferative capacity. 

Hoping that the grafting to dendrimers could increase its anticancer efficiency, ethacrynic acid was modified through the carboxylic acid moiety by peptide coupling with phenolpiperazine, to be grafted to several generations of phosphorhydrazone dendrimers, affording the series of compounds **2-Gn** (n = 1 to 3) (Figure 3) [38]. These dendrimers were tested against solid tumor KB cell line, liquid tumor HL-60 cell line, and non-cancerous quiescent endothelial progenitor cells (EPC). An increase in the efficiency was observed when the number of generations increased, and the third generation was found to be the most efficient. The IC_50_ values (the quantity of dendrimer necessary to kill 50% of the cells) were about 0.1 µM against KB, and 4 µM against HL-60, for the most efficient dendrimer **2-G_3_**. Tests with the EPC cells revealed that above 100 µM of dendrimers was necessary to kill 50% of these non-cancerous cells, which indicates a very good safety ratio for all these dendrimers (see Table in Figure 3).

Ethacrynic acid has been grafted also to phosphorhydrazone dendrimers through another type of linkage, but the anti-cancer properties of these compounds have not been tested [39].

### 2.2. Phosphorhydrazone Dendrimers Functionalized by Metal Complexes 

**Figure 4:**
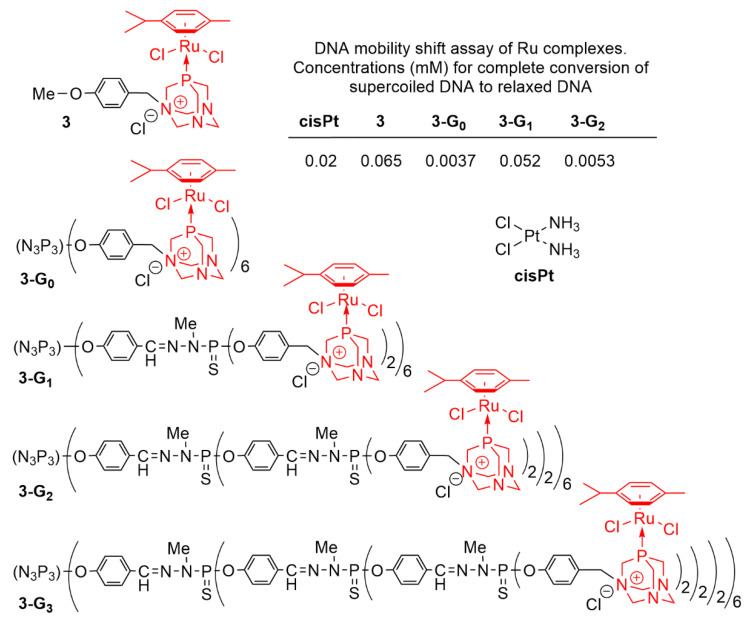
Dendrimers **3-Gn** functionalized by PTA-Ru complexes and their efficiency to convert the supercoiled form of DNA to the relaxed form, compared to cisplatin.

Since the discovery of the properties of cisplatin as an anticancer drug [40], able to bind covalently to DNA with concomitant bending and unwinding of the double helix [41], many other platinum derivatives have been tested as anticancer agents [42]. Since then, the field has been further expanded to the discovery of the anticancer efficiency of other metallic derivatives, for instance based on cobalt, manganese, ruthenium, iron, or copper [43]. Phosphorhydrazone dendrimers functionalized with metal complexes have been first synthesized for catalytic studies [44]. This experience in coordination chemistry led then to the discovery of the anticancer efficiency of several types of dendritic complexes. In some cases, the dendrimer complexes have both catalytic and anticancer properties. This is in particular the case of some dendritic ruthenium derivatives of PTA (phosphatriazaadamantane) [45] pertaining to the **3-Gn** family (Figure 4). These compounds have been successfully used as reusable catalysts in aqueous media for the hydration of alkynes [46] and the isomerisation of allylic alcohols to ketones [47]. The ability of the same dendrimer Ru-complexes **3-Gn** to interact with supercoiled DNA was assayed to detect if this interaction could lead to the relaxed form of DNA, in comparison with the efficiency of cisplatin. The molar concentration at which such phenomenon occurs is shown in the table inside Figure 4. It can be seen that most dendrimers tested have a higher efficiency than cisplatin to lead to the relaxed form of DNA. The dendrimer of generation zero, **3-G_0_**, is the most efficient, whereas the monomer of the same family, compound **3,** is the least efficient [48].

**Figure 5:**
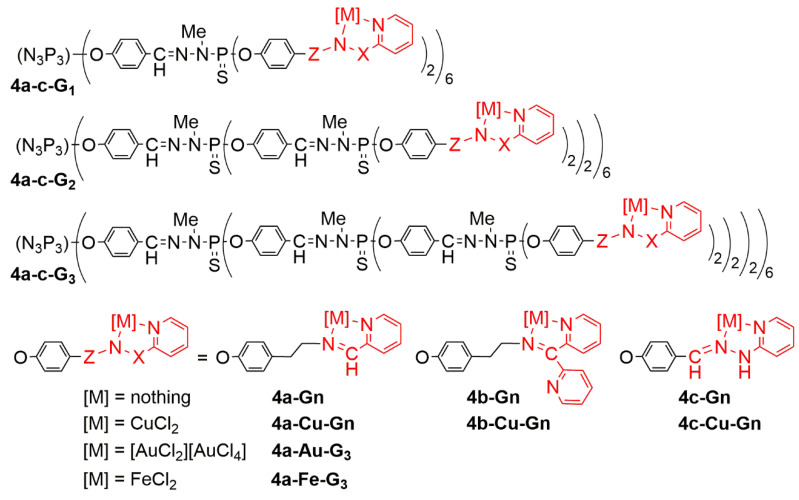
Three generations of dendrimers functionalized by three types of pyridine-imine ligands, and their copper, gold, and iron complexes.

Pyridine-imine copper (I) complexes on the surface of phosphorhydrazone dendrimers have first been used as catalysts in O-arylation reactions, then in the arylation of nitrogen heterocycles, and finally in vinylation of phenol and pyrazole [49]. A series of analogous dendrimers bearing diverse types of pyridine-imine ligands has been synthesized from generation 1 to generation 3 (**4a-c-Gn**), as shown in Figure 5. These dendrimers have been used first for the complexation of copper (II) (CuCl_2_), affording the series of dendrimers **4a-c-Cu-Gn** [50]. All 18 dendrimers synthesized were then tested, i.e., the nine non-complexed dendrimers **4a-c-Gn**, and the corresponding nine copper complexes **4a-c-Cu-Gn**. Tests were carried out against solid tumor KB (epidermal carcinoma) and leukemia HL60 (promyelocytic) cells. The non-complexed dendrimers **4a-Gn** and **4b-Gn** display a potent antiproliferative activity at 10 μM, but largely reduced at 1 μM (excepted for **4a-G_3_**), whereas dendrimers of the **4c-Gn** family do not have any inhibitory effect on cell proliferation. The effect of the generation, and thus of the number of terminal groups, was not noticeable for the **4a-Gn** family, whereas in the **4b-Gn** series, dendrimer **4b-G_3_** was found to be less efficient than dendrimer **4b-G_1_**. Complexation of copper increased the cytotoxicity of the **4c-Gn** family (**4c-Cu-Gn**) at 10 μM, but no activity was observed at 1 μM. An increased toxicity was observed at 1 μM, with the **4b-Cu-Gn** family, compared with the **4b-Gn** family. In the case of the **4a-Cu-Gn** family, a direct relationship between the growth inhibitory effect (% inhibition at 1 μM against HL60 cell line) and the generation of the dendrimer was observed in the copper-complexed series versus the non-complexed series **4a-Gn**. The largest difference was observed with the third generation. Only **4a-Cu-G_3_** displayed a very potent anti-proliferative activity (>80%) at 1 μM against both KB and HL60 cell lines. Importantly, no cytotoxic effect was observed with CuCl_2_ alone (not complexed), nor with the corresponding monomeric copper complexes, at the same concentrations [50]. 

In view of the results of this screening, the most potent dendrimers, the couple **4a-G_3_**/**4a-Cu-G_3_**, was selected for additional investigations against a panel of cancer cell lines including HCT116 (human colon cancer), MCF7 (hormone-responsive breast cancer), OVCAR8 (ovarian carcinoma), and U87 (human glioblastoma-astrocytoma, epithelial-like). In addition, these dendrimers were tested against two non-cancer cell lines, MCR5 (proliferative human lung fibroblasts) and the quiescent EPC (endothelial progenitor cells, Cyprinus carpio). The IC_50_ values determined in this new assay were in the μMolar range between 1.6 and 0.3 μM for **4a-G_3_** and between 0.8 and 0.3 μM for **4a-Cu-G_3_**. This study confirmed the potent anti-proliferative activity of the complexed dendrimer **4a-Cu-G_3_** versus **4a-G_3_** against KB and HL60 cell lines (~2–4 fold improvement). Both dendrimers display similar potency against HCT116, MCF7, and U87 cancer cell lines, showing no difference with or without copper. On the contrary, **4a-G_3_** is about 2-fold more potent than the corresponding **4a-Cu-G_3_** against the OVCAR8 cell line. Interestingly, non-cancer cells (EPC and MCR5) are less sensitive than cancer cell lines to **4a-Cu-G_3_**, contrarily to **4a-G_3_** [50].

To try to understand the reasons of the obtained results, and in particular the large differences observed depending on the type of pyridine-imine ligands and the corresponding complexes, the copper complexes **4a-c-Cu-Gn** were characterized by EPR (electron paramagnetic resonance). Experiments were carried out with either the dendrimer alone, or in the presence of HCT-116 (human colon carcinoma) cell line, and MRC-5, human fetal lung fibroblast normal cells [51]. However in these experiments, it is not the dendritic Cu-complex that is used directly, but the non-complexed dendrimer to which Cu(II) is added in situ. First experiments were carried out with the dendrimer copper complexes alone in DMF (dimethylformamide). It appears that the most stable complex is **4a-Cu-G_3_**, for which the CuN_2_O_2_ coordination, consisting of two nitrogen atoms from the pyridine-imine ligand and two oxygen atoms from the solvent, is predominant. The EPR behaviour of this dendrimer **4a-Cu-G_3_** (also formed in situ) was then investigated in the presence of cells. Results indicate a stronger binding of **4a-Cu-G_3_** with HCT cancer cells with respect to the MRC normal cells, corroborating the lower sensitivity of the normal (EPC and MCR5) cells to **4a-Cu-G_3_**, compared to cancer cells, as indicated just above.

**Figure 6:**
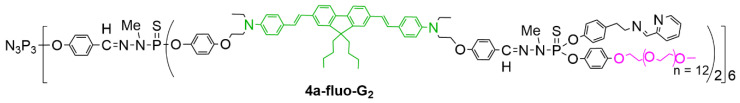
Brightly fluorescent dendrimer, synthesized for investigating the behaviour of the pyridine imine dendrimers in cells.

To further understand the high anti-proliferative potency of dendrimers **4a-G_3_** and **4a-Cu-G_3_**, the biological events induced by these dendrimers were investigated in human cancer KB and HL-60 cell lines, and in the proliferating but non-tumoral MRC5 cell line. In order to ascertain the presence of the dendrimers in different compartment of the cells, a fluorescent analogue was synthesized, namely **4a-fluo-G_2_**. This compound is a second generation dendrimer, bearing 12 highly fluorescent fluorophores at the level of the first generation, having two-photon absorption properties i.e., the ability to absorb simultaneously two photons of lower energy, compared to the classical absorption of one photon [52] (Figure 6). In replacement of half of the pyridine-imine groups, PEG (polyethyleneglycol) derivatives were grafted as terminal functions to increase the solubility, which was decreased by the presence of the fluorophores, constituted of several aromatic groups. This dendrimer **4a-fluo-G_2_** avidly binds to the cell membrane, even after cell washes. After 24 h, **4a-fluo-G_2_** has entered the intracellular space by endocytosis in a high proportion. It was then shown that the dendrimers induced cell death through the activation of the apoptotic process. Apoptosis is a programmed cell death, tightly regulated via the activation of cellular proteases leading to the cleavage of chromatin into nucleosomal fragments, in contrast to necrosis, which involves the destruction of the plasma membrane leading to the release of cytosolic enzymes and cofactors into the external medium. 

Caspase-3 (cysteine-aspartic acid protease) is the major contributor to cellular DNA fragmentation in the apoptosis process. Dendrimer **4a-G_3_** markedly stimulated the activity of caspase-3 in KB cells, but had no effect in HL-60 cells. Unexpectedly, dendrimer **4a-Cu-G_3_** significantly reduced the activity of caspase-3 in both types of cells. Another experiment indicated that dendrimers **4a-G_3_** and **4a-Cu-G_3_** promote the translocation of proapoptotic proteins from the endoplasmic reticulum to the nuclei, to participate in DNA fragmentation, which is a hallmark for apoptosis, dendrimer **4a-Cu-G_3_** being more active than **4a-G_3_**. In addition, dendrimer **4a-Cu-G_3_** is more potent than **4a-G_3_** to promote the translocation of Bax (a pro-apoptotic protein, major contributor for the opening of pores into the mitochondrial membrane), from cytosol where it is quiescent to mitochondria where it is active. In sharp contrast with cisplatin, these phosphorus dendrimers complexed or not with Cu play a protective anti-oxidant role in cells, by decreasing the production of ROS (reactive oxygen species), and they do not alter the cell cycle, emphasizing a totally different mechanism of action. Thus, dendrimer **4a-Cu-G_3_** is the first member of a new class of promising anti-proliferative agents, with a distinctive mode of action [53].

**Figure 7:**
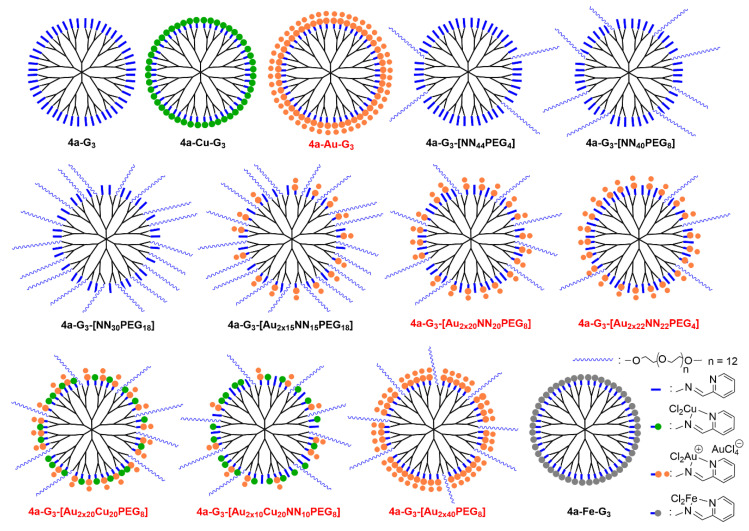
Schematization of the functionalization of the third generation dendrimer. For dendrimers obtained by the stochastic functionalization of the surface, only one of the innumerable possible structure is shown. The names in red correspond to the most active dendrimers.

In order to expand this new class of anti-proliferative agents, it was found interesting to modify the type of metal complexed. In a first attempt, 48 equivalents of AuCl_3_ were added to the dendrimer **4a-G_3_** but only half of the terminal functions were complexed. In fact, 96 equivalents of AuCl_3_ were necessary to complex all the 48 pyridine-imine functions of the dendrimer **4a-G_3_**. Indeed, each pyridine-imine group did not complex AuCl_3_ but AuCl_2_^+^, with AuCl_4_^-^ as counterion, as shown in Figure 5 for dendrimer **4a-Au-G_3_**, and as schematized in Figure 7. This dendrimer is active (IC_50_) in the low nanomolar range against both KB and HL-60 cancer cell lines, that is about two orders of magnitude better than the corresponding copper complexes. Furthermore, the IC_50_ towards the non-cancerous (quiescent) cell line EPC is higher than 1000 nM; this means that the safety ratio is very good with the gold complexes (see Table 1).

In order to determine the influence of the number of gold moieties on the surface of the dendrimers towards the proliferative activities, nine new dendrimers have been synthesized by the stochastic functionalization of the surface of the third generation dendrimer. A variable number of free pyridine-imine ligands, copper complexes, gold complexes, and PEG derivatives (13 CH_2_CH_2_O linkages) have been grafted to the surface of this dendrimer. One of the possible structures for each of these stochastically functionalized dendrimers is schematized in Figure 7 [54]. Dendrimers having only non-complexed pyridine-imine ligands and PEG groups in variable proportion displayed no activity against the cancerous and non-cancerous cell lines at least at 1000 nM (Table 1). For functionalizations with mixtures of free ligands, gold complexes, and PEGs, a large number of PEG derivatives has a detrimental influence on the activity, probably because the gold complexes are screened by the PEGs and cannot exert their cytotoxic activity, as shown for instance by the low activity of compound **4a-G_3_-[Au_2x15_NN_15_PEG_18_]** (Table 1). However, the presence of a few PEG derivatives is beneficial for the activity, and dendrimer **4a-G_3_-[Au_2x40_PEG_8_]** is the most active of all the series. In order to determine if a synergistic effect could be observed between copper and gold, two dendrimers complexing both metals, as well as a few PEGs, and eventually free ligands have been synthesized. Both compounds are very active, even the one having only 10 gold complexes, but the activity is not better than without copper. So the high activity is provided by gold, only 10 gold complexes among 48 terminal functions are sufficient, and further increasing the number of gold complexes did not really improve the activity.

In a last experiment in this series of dendrimers, iron was complexed on the surface of the third generation dendrimer instead of copper or gold (compound **4a-Fe-G_3_** in Figure 7). No activity was observed below 1000 nM against both the cancerous and non-cancerous cell lines, thus this iron complex is not a suitable anti-cancer agent [55]. Few derivatives of iron have demonstrated anti-cancer activity and they are essentially based on ferrocenyl (metallocene) derivatives [56].

**Figure 8:**
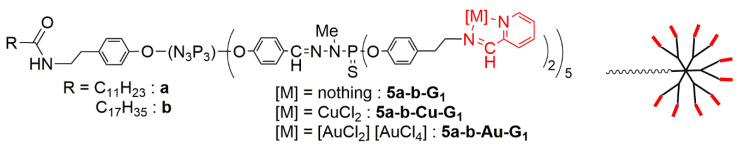
First generation off-center dendrimers bearing one alkyl chain at the core and 10 pyridine-imine ligands as terminal groups, either free or complexing copper or gold.

**Figure 9:**
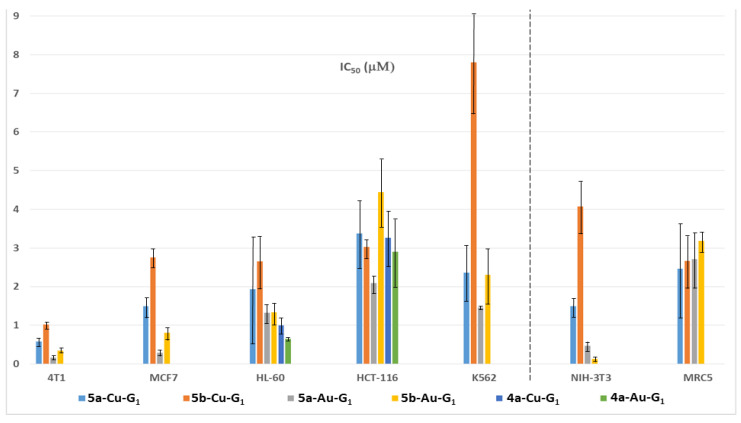
IC_50_s of four off-center dendrimers (series 5) and two dendrimers (series 4) towards five cancerous cell lines and two non-cancerous (NIH-3T3 and MRC5).

For further expanding the scope of this study, after changing the type of ligands and of metals, the shape of the dendrimer was changed by synthesizing off-center dendrimers [57], which also could be called dendrons [58]. These compounds were synthesized from the cyclotriphosphazene core [59], as were the dendrimers, but only five functions among six were used for the growing of the dendrimer [60], the sixth one being used for the grafting of an alkyl chain. The selective functionalization of cyclotriphosphazene [61] is indeed a very powerful tool for the synthesis of specific dendritic structures [62], suitable in particular for biological purposes [63]. As the alkyl chain should be entirely entrapped inside a third generation dendrimer [64], only the first generation off-center dendrimers were synthesized, as shown in Figure 8. The pyridine-imine ligands were the same as for the dendrimers, and both the copper and gold complexes were studied. Two alkyl chain lengths were used: C_11_H_23_ and C_17_H_35_. These off-center dendrimers were tested against a panel of two aggressive breast cancer cell lines (4T1, mouse breast adenocarcinoma cells and MCF-7, human breast adenocarcinoma cells), three other cancer cell lines (leukemia HL-60, human colon cancer HCT-116, and the chronic myeloid leukemia cell line K562), and two non-cancerous (normal fibroblast NIH-3T3 and human fetal lung fibroblast cells MRC5), and were compared in some cases with the corresponding first generation dendrimers (Figure 9). Attempts were carried out with the non-complexed off center dendrimers **5a-G_1_** and **5b-G_1_**, but they were rapidly discarded as being non-active at 100 µM. The longest alkyl chain (series **5b**) has a detrimental effect on the efficiency in most cases. The gold complexes are generally more efficient than the copper ones, but the difference is not large compared to what was observed with the third generation dendrimer Cu and Au complexes, but with other cell lines. The mechanisms of action are relatively similar to those elucidated with dendrimers. However, contrarily to dendrimers, the gold complexes of the dendrons are highly toxic against the non-cancerous cell line NIH-3T3 [65].

## 3. Phosphorus Dendrimers as Carriers of Anticancer “Drugs” 

**Figure 10:**
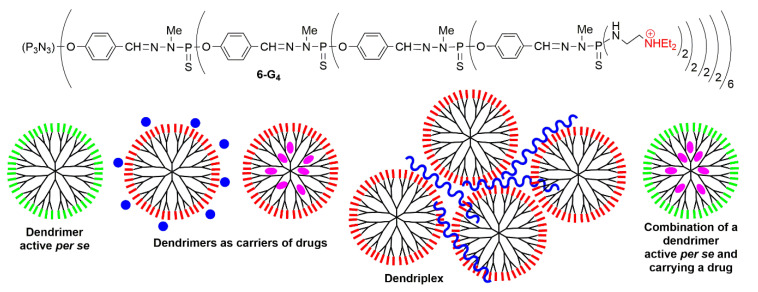
Positively charged dendrimer **6-G_4_**, used as carrier.

The usefulness of positively charged dendrimers as non-covalent carriers was recognized very early for transfection experiments [66]. Positively charged phosphorus dendrimers, functionalized by triethylammonium terminal groups, have been found useful not only in biology, but also in materials chemistry for the elaboration of nanotubes [67,68] or microcapsules made of dendrimers [69] and the functionalization of silica [70] or clays [71]. However, they were used first as carriers of the luciferase plasmid, helping its penetration into 3T3 cells. It was shown that the efficiency increased with the generations on-going from the first to the third generations, then a plateau was reached with the fourth (**6-G_4_**, Figure 10) and fifth generations. These dendrimers were more efficient in the presence of serum than without and were as efficient as one of the chemical standards for transfection, i.e., PEI (polyethyleneimine) [72]. The same family of dendrimers was then used to deliver fluorescein-labeled oligodeoxyribonucleotide and a DNA plasmid containing the functional gene of enhanced green fluorescent protein (EGFP) [73] into HeLa cells [74]. The ammonium terminal functions were then modified to incorporate in particular cyclic ammonium groups, such as pyrrolidine, morpholine, and piperazine [75]. These families of positively charged phosphorus dendrimers have been used as drugs by themselves against prion diseases (transmissible spongiform encephalopathies), including the BSE (Bovine spongiform encephalopathy, also called mad cow disease), both in vitro and in vivo [23]. They are also able to decrease the aggregation of peptide Aβ 1-28 and of the Map-Tau protein, both involved in the Alzheimer disease [24]. These families of positively charged phosphorus dendrimers have been used after as carriers of anticancer drugs, of anticancer siRNA, and in other combinations, as shown in Figure 10, and as will be displayed below. Indeed, the hydrophilic surface and hydrophobic backbone of phosphorus dendrimers made them suitable tools to penetrate membranes [76].

### 3.1. Phosphorhydrazone Dendrimers as Carriers of Known Anticancer Drugs 

Dendrimer **6-G_4_** has been used as carrier of 8-anilino-1-naphthalenesulfonate as a fluorescent model of drugs. Two binding sites were identified: deep inside the dendrimer, and close to or on the surface of the dendrimer. The same dendrimer was then used as carrier of the cytostatic drug cisplatin (see Figure 4 for its structure). The presence of the dendrimer greatly increased the efficiency of cisplatin towards cell cultures of craniospinal cancer of the fourth ventricle (IV stage). Indeed, 1 µg/mL of cisplatin alone induced a cytotoxicity of 61.9 ± 8.8%, whereas a 10-fold lower quantity of cisplatin (0.1 µg/mL) entrapped in 1 µg/mL of dendrimer **6-G_4_** induced a higher cytotoxicity (74.1 ± 4.1%). Thus, this dendrimer greatly enhances the cytotoxicity of cisplatin towards a brain tumor [77]. 

**Figure 11:**
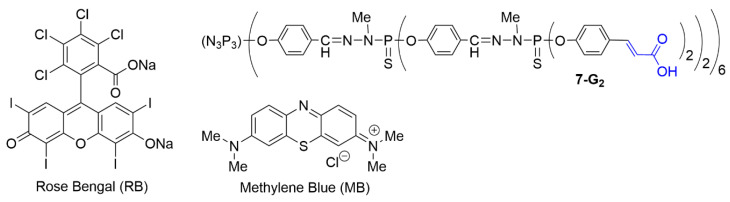
Chemical structure of the photosensitizers Rose Bengal and Methylene Blue, and of dendrimer **7-G_2_**.

Dendrimer **6-G_3_** was used to carry by electrostatic interactions the negatively charged photosensitizer Rose Bengal (RB, Figure 11), used in particular in photodynamic therapy [78], through the generation of singlet oxygen. RB has a strong tendency to aggregate in water, thus losing a large part of its activity [79]. Complexing RB with a dendrimer may significantly prevent its aggregation in water. About 7 RB molecules were interacting per phosphorus dendrimer. It was shown that the RB-**6-G_3_** complex generated significantly more singlet oxygen than did free RB. The penetration of RB alone or RB-**6-G_3_** complex was measured in three murine basal cell carcinoma lines (ASZ, BSZ, and CSZ). A largely higher uptake was observed in the case of the RB-**6-G_3_** complex. In the absence of light, the viability of these cells was not affected by the presence of RB or RB-**6-G_3_**. However, upon wide range irradiation (visible light from 385–780 nm), a large difference in efficiency was observed between RB and RB-**6-G_3_** for the three cell lines. For example, for a concentration of 0.5 μM in RB, in the case of ASZ cells, for RB alone the cell viability was very high (90%), whereas for RB-**6-G_3_** the cell viability was decreased to only 7%, due to an enhanced ^1^O_2_ production [80].

Methylene blue (MB) is another type of photosensitizer, but contrarily to Rose Bengal, it is positively charged. However, it is also prone to aggregation and to rapid chemical alterations in a biological environment, inducing negligible photodynamic efficiency [81]. Its association with a dendrimer to possibly increase its stability can be carried out only with a negatively charged dendrimer. The second generation dendrimer **7-G_2_**, bearing 24 carboxylic acid terminal functions, was used for this purpose (Figure 11). This dendrimer was first synthesized for carrying n-hexadecylamino-1-deoxylactitol to mimic galactosylceramide (Galβ1cer), with the goal of blocking HIV infection prior to the entry of the virus into human cells [82]. The MB-**7-G_2_** complex (5 MB molecules per dendrimer) was found to generate less singlet oxygen than MB alone. However, tests with basal cell carcinoma cell lines (ASZ, BSZ, and CSZ) revealed a higher cellular uptake. Upon irradiation with visible light, phototoxicity against basal cell carcinoma cell lines was increased, accompanied with enhanced production of ROS [83].

### 3.2. Phosphorhydrazone Dendrimers as Carriers of Therapeutic Oligonucleotides

Small interfering RNAs (siRNAs) are a class of double-stranded RNA, non-coding RNA molecules, of generally 20–25 base pairs in length, which induce selective gene silencing. Depending on their structure, siRNAs can be used for the manipulation of apoptosis, to force specific cells to die, in particular cancerous cells [84]. Phosphorus dendrimers **6-G_3_** and **6-G_4_**, PAMAM dendrimers of generations 3 and 4, and carbosilane dendrimers of generation 2 were used as carriers of three anticancer siRNAs directed against anti-apoptotic proteins of the BCL family, at a P/N (phosphates of SiRNA to ammoniums of the dendrimers) ratio of 3.33/1 in all cases. Transfection of the complexes was carried out in two cancerous cell lines HeLa and HL-60. Complexes based on dendrimers **6-G_3_** and **6-G_4_** with all the selected siRNAs were taken up by cells more efficiently than complexes based on all the other dendrimers. Experiments carried out with a mixture of the three siRNAs induced a significant decrease in the viability of the cells, in particular when using the phosphorhydrazone dendrimers as carriers [85].

Dendrimers **6-G_3_** and **6-G_4_** and PAMAM dendrimers generations 3 and 4 have been used also as carriers of the siRNA of polo-like kinase (siPLK1) [86], as PLK1 has been reported as a potential target for triple negative breast cancer (TNBC) [87], which is irresponsive to common treatments [88]. Indeed, siPLK1 is reported to arrest the cells in sub-G1 phase (marker of apoptosis). Dendriplexes were formed at 3:1 N/P ratio for all dendrimers and induced enhanced cell uptake of siPLK1 compared to siPLK1 solution in MDA-MB-231 and MCF-7 cells, which are two types of TNBC cell lines. Cycle arrest in sub-G1 phase was observed with all dendriplexes. No significant difference on the efficiency was observed between the dendriplexes formed with PAMAM and phosphorus dendrimers generations 3 and 4 [89]. 

**Figure 12:**
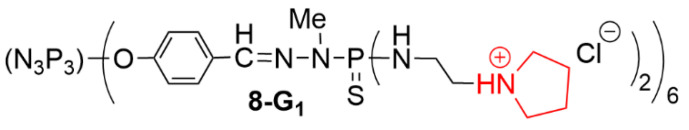
First generation dendrimer bearing pyrrolidinium terminal functions.

The best generation to be used for transfection experiments remains an open question, as shown before. Generations 1 to 3, bearing as terminal functions cyclic ammonium derivatives, were tested as carriers of plasmid DNA (pDNA), encoding the enhanced green fluorescent protein (EGFP). It was shown that the first generation bearing 1-(2-aminoethyl) pyrrolidinium as terminal functions (dendrimer **8-G_1_** [75], Figure 12) was the most efficient. This dendrimer **8-G_1_** was then applied as carrier of pDNA-p53 (plasmid DNA encoding both EGFP and the tumor suppressor p53 protein). A significant p53 protein expression was observed in HeLa cells. The cancer gene therapy potential of the dendriplex **8-G_1_**/pDNA-p53 was then validated through therapy of a xenografted tumor-bearing mice after intra-tumoral injection [90].

### 3.3. Associations of Therapeutic Agents against Cancers

**Figure 13:**
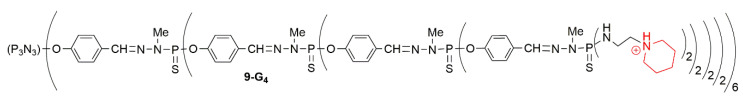
Generation 4 dendrimer used as a carrier of both 5-fluorouracil and a mixture of anti-cancer siRNAs.

It has been shown in the two previous paragraphs (3.1 and 3.2) that phosphorus dendrimers can be used for delivering either anti-cancer drugs or siRNA. An attempt has been made to combine both approaches, expecting a synergistic effect. A generation 4 phosphorhydrazone dendrimer functionalized with piperidinium terminal functions (**9-G_4_**, Figure 13) has been used as a vector for both the chemotherapeutic agent 5-fluorouracil (5-FU) [91] and a mixture of anti-cancer siRNAs able to downregulate anti-apoptotic genes (BCL-xL, BCL-2, MCL-1) [92]. The cytotoxic effect was evaluated on human cervical carcinoma cells (HeLa cell line). A considerable increase of 5-FU cytotoxic effect was observed by addition of the **9-G_4_**/siRNA cocktail dendriplexes in low doses [93].

Another example of association of therapeutic agents concerned the dendritic copper complex **4a-Cu-G_3_** associated with five cytotoxic agents used in chemotherapy (cisplatin, camptothecin, paclitaxel, doxorubicin, and MG132, which is a synthetic peptide aldehyde [94]). Different results were obtained depending on the type of drug and especially on their modes of action. No additive effect was observed with camptothecin and cisplatin, but it was observed with paclitaxel and MG132. More interestingly, synergy was observed with doxorubicin [95].

## 4. Indirect Anticancer Activity of Phosphorus Dendrimers

In this section are gathered a few examples of phosphorhydrazone dendrimers that are not cytotoxic by themselves, but which induce a cytotoxic effect on cancerous cells.

**Figure 14:**
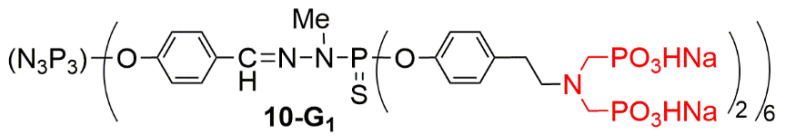
First generation dendrimer capped with azabisphosphonate functions.

The first example concerns a first generation dendrimer capped with 12 azabisphosphonate terminal functions (**10-G_1_**, Figure 14), which is able to trigger the human immune system towards an anti-inflammatory process, is efficient against chronic (rheumatoid arthritis [27], neuro-inflammation [96], or psoriasis [97]) and acute inflammatory diseases (Uveitis) [28]. Among other properties, this compound is able to multiply by several hundreds the number of Natural Killer (NK) cells [98] after 3 weeks in culture, starting from human PBMCs (Peripheral Blood Mononuclear Cells). NK cells are a very important component of the human immune system, as they are able to fight against viral and bacterial infections, but also against numerous types of cancers. The NK cells produced thanks to the dendrimer were tested against seven leukemia and seven carcinoma types of cell lines. They were found efficient against all these cancerous cells, especially against the K562 leukemia, which is one of the main targets of NK cells [99]. A complex mechanism, not yet fully understood, explains the proliferation of the NK cells. The first step is the anti-inflammatory activation of monocytes in 3 days [100], followed by the inhibition in 1 week of the proliferation of CD4+ T cells, without affecting their viability [101]. In fact, this dendrimer induces a complex equilibrium between the anti-inflammatory activities, through the activation of monocytes, and the anti-cancer properties, through the multiplication of NK cells [102]. Surprisingly, the internal structure of this dendrimers plays a crucial role in the activity [103].

**Figure 15:**
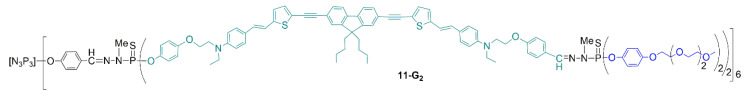
Fluorescent phosphorhydrazone dendrimer potentially useful for photodynamic therapy under two-photon irradiation.

A last example of indirect influence of a dendrimer concerns a second generation dendrimer having short PEG derivatives on the surface, and, inside the structure, a specially engineered fluorophore, able to be excited classically by the absorption of one photon, but also by the absorption of two photons simultaneously [104] (Figure 15). Other types of phosphorus dendrimers possessing in their structure fluorophores having two-photon absorption (TPA) properties have been synthesized previously [105,106], and used in particular for imaging in vivo the blood vessel of a rat olfactory bulb [107]. Besides its fluorescence properties, dendrimer **11-G_2_** is also able to generate singlet oxygen under two-photon irradiation. It has been introduced in cultures of MCF-7 human breast cancer cells, in which it is internalized easily after 3 h of incubation. This dendrimer was found non-toxic in the dark, but also under daylight irradiation. On the contrary, under TPA conditions (irradiation at 760 nm for 3 × 1.57 s), this dendrimer is highly toxic for these cancerous cells. Thus, this dendrimer combines bioimaging properties with photodynamic therapy properties [108].

## 5. Conclusions

In this review, we intended to demonstrate the usefulness of phosphorhydrazone dendrimers to fight against cancers, through several types of approaches. Most of the experiments have been carried out in vitro on cancerous cell lines, but a few studies have been carried out in vivo, in particular the therapy of a xenografted tumor-bearing mice [90]. Some of these dendrimers, in particular some gold complexes, are very active at the low nanomolar range, with an excellent safety ratio towards non-cancerous cells, and should deserve further studies. Furthermore, some of these phosphorhydrazone dendrimers are potentially useful for combining diagnostics and therapy, i.e., for the theranostic [109]. Taken altogether, the phosphorhydrazone dendrimers, suitably functionalized, are indeed useful potential nanotools against cancers. However, the translation from the bench to the bedside is an important challenge for dendrimers. Only a very limited number of clinical trials have been carried out to date with dendrimers. One can cite in particular Phase III clinical trials for the treatment of bacterial vaginosis, using VivaGel^®^ (SPL7013) from Starpharma [110], which is a generation 4 polylysine dendrimer, ended by a 2-[(3,6-disulfo-1-naphthalenyl)-oxy] acetic acid disodium salt. A polylysine dendrimer is in Phase II clinical trial as a nanocarrier for encapsulating docetaxel, this association showing superior anticancer activities against several types of solid cancers [111]. Now, the main challenge for dendrimers, including for phosphorus dendrimers, is to jump the “valley of death” between research and clinical applications.

## Figures and Tables

**Table 1:** IC_50_ values (nM) and safety ratios (IC_50_ EPC/IC_50_ KB and IC_50_ EPC/IC_50_ HL-60).

Dendrimer	KB	HL-60	EPC	EPC/KB	EPC/HL-60
**4a-G_3_**	1600 ± 150	1300 ± 100	360 ± 200	0.225	0.277
**4a-Cu-G_3_**	470 ± 20	580 ± 70	800 ± 180	1.7	1.4
**4a-Au-G_3_**	7.5 ± 7.5	3.3 ± 0.6	>1000	>133	>303
**4a-G_3_-[NN_44_PEG_4_]**	>1000	>1000	>1000		
**4a-G_3_-[NN_40_PEG_8_]**	>1000	>1000	>1000		
**4a-G_3_-[NN_30_PEG_18_]**	>1000	>1000	>1000		
**4a-G_3_-[Au_2x15_NN_15_PEG_18_]**	87 ± 7	>1000	>1000	>11.5	
**4a-G_3_-[Au_2x20_NN_20_PEG_8_]**	15 ± 5	4.5 ± 0.5	>1000	>67	>222
**4a-G_3_-[Au_2x22_NN_22_PEG_4_]**	6.7 ± 4.6	3 ± 0.5	>1000	>149	>333
**4a-G_3_-[Au_2x20_Cu_20_PEG_8_]**	8.5 ± 0.7	2.5 ± 0.7	>1000	>118	>400
**4a-G_3_-[Au_2x10_Cu_20_NN_10_PEG_8_]**	10 ± 3	4 ± 3	>1000	>100	>250
**4a-G_3_-[Au_2x40_PEG_8_]**	5.5 ± 0.5	1.7 ± 0.5	>1000	>182	>588
**4a-Fe-G_3_**	>1000	>1000	>1000		
